# Cold-adaptive variations in the membrane lipid composition in polar species of the ciliate *Euplotes*

**DOI:** 10.3389/fmicb.2026.1909674

**Published:** 2026-08-24

**Authors:** Andrea Anesi, Claudio Alimenti, Pierangelo Luporini, Graziano Guella, Adriana Vallesi

**Affiliations:** 1 Unit of Metabolomics, Department of Food Quality and Nutrition, Research and Innovation Center, Fondazione Edmund Mach (FEM), San Michele all'Adige, Italy; 2 Laboratory of Eukaryotic Microbiology and Animal Biology, School of Biosciences and Veterinary medicine, University of Camerino, Camerino, Italy; 3 Laboratory of Bioorganic Chemistry, Department of Physics, University of Trento, Trento, Italy

**Keywords:** Antarctic ciliates, cold adaptation, *Euplotes*, membrane lipids, survival strategies, polar biology

## Abstract

Thriving in the freezing polar seas forces organisms, in particular the unicellular ones directly exposed to the extracellular environment, to adaptively modify the lipid composition of their cellular membranes in function of maintaining an appropriate degree of fluidity. To seek into these modifications, we carried out a comparative analysis of the membrane lipid composition in four marine species of the globally distributed ciliate, *Euplotes*. Two, *E. focardii* and *E. nobilii*, are polar species that differ ecologically and physiologically from one another. The former is endemic to the Antarctic coastal waters and strictly psychrophilic (it does not survive over 10 °C), while the latter is characterized by a bipolar distribution and a psychrotrophic (rather than psychrophilic) behavior (it reproduces up to 15 °C). The two other species, *E. crassus* and *E. raikovi*, are widespread in temperate seas and phylogenetically closely related, the former, to *E. focardii* and, the latter, to *E. nobilii*. With respect to their temperate-water relatives, *E. focardii* and *E. nobilii* appear to similarly rely on significantly increased concentrations of lipid molecules with higher unsaturation indices and a conical shape to maintain an appropriate degree of membrane fluidity and curvature. Nevertheless, they markedly differ from one another in the membrane lipid composition. In the *E. focardii* membranes, a single lysophosphatidylcholine molecular species accounts for 17.8% of total glycerophospholipids and only 118 structurally distinct lipid molecules are identified, of which 38 are phosphatidylcholines and eight are phosphatidylethanolamines. In the *E. nobilii* membranes, phosphatidylethanolamines represent 47.3% of total glycerophospholipids and 226 distinct lipid molecules are identified, of which 115 are phosphatidylcholines and 55 are phosphatidylethanolamines. These functionally significant differences in the lipid membrane composition between the two polar species likely reflect a more stringent cold-adaptation strategy evolved by *E. focardii* which is exposed to a constantly subzero temperature in relation to its Antarctic endemism, and a more flexible strategy adopted by *E. nobilii* to face more variable temperatures in relation to its bipolar distribution and trans-equatorial migration via the deep ocean currents.

## 1 Introduction

Polar marine ecosystems host a huge variety of colonizers adapted to face unique, extreme environmental conditions. Year-round freezing temperatures impose structural and functional modifications of protein and lipid molecules playing key roles in basic metabolic pathways and mechanical properties of the cell membranes. Pushed by biotechnological perspectives, modifications of proteins with signaling and enzymatic activities have in general attracted more research interest (Van der Berg, 2003; Xu et al., 2024; Alimenti et al., 2009; Tutino et al., 2009; Yang et al., 2013; Santiago et al., 2016; Singh et al., 2016; Parvizpour et al., 2021). Investigations on modifications of membrane lipids have, instead, been motivated mainly by a curiosity-driven interest to know how polar organisms preserve a proper fluidity of their cell membranes and prevent cold-induced increases of the lipid intermolecular hydrogen bonding responsible for the cell membrane transition from the (so-called) “liquid-crystalline phase” to the “gel phase” (Suutari and Laakso, 1994; Beales, 2004; Leekumjorn and Sum, 2007). Varied numbers of polar species of bacteria (Russell, 1997; De Maayer et al., 2013; Guan et al., 2013; De Maayer et al., 2014; Hassan et al., 2020), yeasts (Rossi et al., 2009; Klose et al., 2012; Kelso et al., 2025), algae (Smith et al., 1993; Henderson et al., 1998; Thomson et al., 2004; Teoh et al., 2013), dinoflagellates (Nichols et al., 1993; Leblond et al., 2006; Gray et al., 2009; Flaim et al., 2012, 2014; Anesi et al., 2016), fungi (Hammonds and Smith, 1986; Singh et al., 2006) and animals (Palmerini et al., 2009; Hagen et al., 2000; Mayzaud et al., 2011; Malekar et al., 2018; Trenti et al., 2022) have been analyzed for cold-induced modifications of their membrane lipid composition. Experimental evidence converges on identifying fatty acids as the primary target of these modifications, in the first place represented by increased levels of unsaturation, alterations in the position and conformation of the double bonds in the hydrocarbon chains, and changes in the length of the acyl chains (Suutari and Laakso, 1994; Chintalapati et al., 2004; Morgan-Kiss et al., 2006; Rodrigues and Tiedje, 2008; Ernst et al., 2016).

To improve the knowledge of the cold-adaptive modifications of membrane lipid composition in polar eukaryotic microorganisms, we thought of ciliates as a particularly promising research material for two major experimental reasons. First, their abundance in polar species implies an intrinsic structural and functional cell plasticity to promptly adapt to freezing temperatures (Wickham et al., 2011; Jiang et al., 2014). Second, their direct interactions with the extracellular environment are mediated through an incomparably extended plasma membrane, uplifted to wrap vast populations of cilia functionally diversified between single sensory bristle-cilia and complex locomotory organelles known as cirri and membranelles (Hausmann and Radek, 2014).

This work reports a comparative analysis of the membrane lipid composition in four congeneric marine species of *Euplotes*, an extremely diversified and cosmopolitan ciliate (Di Giuseppe et al., 2015), all of which have long been investigated in particular for the genetics and structural biology of the self/not-self mechanism that controls their reproduction and sex realized in the form of conjugation (Luporini et al., 2016; Vallesi et al., 2016; Alimenti et al., 2022). Two, *E. focardii* and *E. nobilii*, thrive in polar waters, and the other two, *E. crassus* and *E. raikovi*, in temperate waters. The former is phylogenetically more closely related to *E. focardii*; the latter, to *E. nobilii* (Di Giuseppe et al., 2015; Syberg-Olsen et al., 2016; Alimenti et al., 2022, 2025). Although sharing a polar habitat, *E. focardii* and *E. nobilii* differ from one another in relation to relevant ecological and cold-adaptive traits. The former is a strictly psychrophilic species endemic to the Antarctic coastal waters constantly recording a subzero temperature (Valbonesi and Luporini, 1990a, 1993). It bears transcriptionally silent heat-shock genes and rapidly collapses at temperatures higher than 8–9 °C (La Terza et al., 2001). The latter, instead, is a psychrotrophic (rather than psychrophilic) bipolar species that dwells in the Arctic seas besides that in Antarctic waters, and bears transcriptionally fully active heat-shock genes withstanding temperatures of 13–15 °C (La Terza et al., 2001). Its antipolar populations maintain a common gene pool by trans-equatorial dispersing via the deep cold ocean currents (Di Giuseppe et al., 2011, 2013).

While showing a similar degree of lipid unsaturation higher than in their temperate-water relatives, *E. focardii* and *E. nobilii* markedly differ from one another in their membrane lipid composition. In the former, it appears to be characterized by a significantly high concentration of a single lyso-phosphatidylcholine molecular species, and a reduced total number of structurally distinct molecular species evenly distributed across different classes; in the latter, by an increased amount of phosphatidylethanolamines and a markedly high number of structurally distinct lipid molecular species unevenly distributed across different classes.

## 2 Materials and methods

### 2.1 Chemicals

Chemicals and solvents for lipid purification and analyses were purchased from Merck (KGaA, Darmstadt, Germany).

### 2.2 Cell cultures

Cell cultures for lipid analysis were expanded from stably cultivated wild-type strains using, as standard food, the green alga *Dunaliella tertiolecta* grown in pasteurized natural seawater (salinity, 32–33 ‰; pH, 8.1–8.2) enriched with Walne medium. The strains TN_1_ (GenBank EF094961.1) and AC_1_ (GenBank GU479385.1) chosen to represent *E. focardii* and *E. nobilii*, respectively, were constantly maintained in a cold room set up at 2–4 °C. Both strains were raised from specimens collected from coastal sites in the proximity of the Italian research station at Terra Nova Bay (Ross Sea, Antarctica) (Valbonesi and Luporini, 1990b; Valbonesi et al., 1992), The strains L-2D (GenBank AJ305255) and TM_3_ (GenBank AJ305251) chosen to represent *E. crassus* and *E. raikovi*, respectively, were instead maintained at a room temperature of 20–22 °C. They were raised from specimens collected, the former, from a site of the Tyrrhenian coast of Italy (Valbonesi et al., 1992) and, the latter, from a site of the Adriatic coast (Miceli and Luporini, 1981). For each lipid extraction, 5-liter cultures, at a density of approximately 5,000 cell/ml, were used.

### 2.3 Lipid extraction

Cells were processed after having been starved for 3–6 days to eliminate any trace of food-derived lipids within digestive vacuoles and ensure that analyses fully reflected the genuine membrane lipid composition. Risks of inter-specific variations consequent to some degree of starvation-induced membrane lipid remodeling were minimized by rigorously following in all the four species the same Folch-protocol of lipid extraction (Folch et al., 1957). Cell pellets were placed into 15 ml-glass tubes, resuspended in 10 ml of chloroform/methanol 2:1 (v/v), sonicated for 15 min in an ultrasonic bath (Sonorex Super, Bandelin electronics, Berlin, Germany), and centrifuged at 3,000 x g for 10 min at room temperature to separate the organic phase (bottom layer). The lipid extraction was repeated three times, and the obtained organic phases were pooled, filtered under vacuum using glass filters, dried on a rotary evaporation apparatus (Büchi Labortechnik AG, Flawil, Swiss) and finally resuspended in 650 μl of deuterated methanol (CD_3_OD) (Merck KGaA, Darmstadt, Germany) for subsequent analyses.

### 2.4 Nuclear magnetic resonance analysis

Lipid extracts were subjected to ^1^H-Nuclear Magnetic Resonance (NMR) (400 MHz) and ^31^P-NMR (162 MHz) analysis on a Bruker-Avance 400 MHz NMR spectrometer, using a 5 mm Broadband Inverse probe equipped with pulsed-gradient field utility. The ^1^H-90° proton pulse length was 9.3 μs with a transmission power of 0 dB, while the ^31^P-90° proton pulse length was 17 μs with a transmission power of −3 dB. Spectra were acquired at 300 K with the chemical shift scale (δ) calibrated on the residual proton signal of CD_3_OD at δ_H_ 3.310 ppm and the ^31^P-NMR δ scale calibrated on phosphatidylcholine (PC) signal at δ_P_ −0.550 ppm. The recorded two-dimensional NMR spectra included proton–proton scalar correlation (^1^H–^1^H DQCOSY), proton–carbon single-bond correlation (^1^H–^1^^3^C HSQC) and proton–carbon multiple-bond correlation (^1^H–^1^^3^C HMBC). One-dimensional NMR spectra were fitted and integrated using MestreNova 8.1 software (MestreLab Research S.L., Escondido, CA). The lipid classes were assigned through comparisons to lipid standards and the relative molar ratios of the lipid classes were determined by integrating the ^1^H NMR signals. Abbreviations of the identified lipid classes are reported in Table 1.

**Table 1:** Classification and abbreviations of lipids identified in *Euplotes* membranes, based on the LIPID MAPS classification system.

Category	Class	
Fatty acyls	Fatty acids (FAs)	Saturated (SFA)
		Monounsaturated (MUFA)
		Polyunsaturated (PUFA)
Glycerophospholipids (GPLs)	Phosphatidylcholine (PC)	
	Phosphatidylethanolaminee (PE)	
	Phosphatidylinositol (PI)	
	Phosphatidylglycerol (PG)	
	Lysophosphatidylcholine (LPC)	
	Lysophosphatidylethanolamine (LPE)	
Sphingolipids	Sphingomyelin (SM)	
Betaina lipids (BLs)	Diacylglyceryltrimethylhomoserine (DGTS)	
	Diacylglycerylhydroxymethyltrimethyl-b-alanine (DGTA)	
	Monoacylglyceryltrimethylhomoserine (LDGTS)	
	Monoacylglycerylhydroxymethyl-b-alanine (LDGTA)	
Saccharolipids	Sulfoquinovosylglycerol (SQDG)	

### 2.5 Molecular species assignment

Following protocols refined by Anesi et al. (2016) and Anesi and Guella (2015), the separation of lipid molecular species was carried out using two complementary Liquid Chromatography-Mass Spectrometry (LC-MS) analyses: Reverse Phase Liquid Chromatography-ElectroSpray Ionization-Ion Trap-Mass Spectrometry (RPLC-ESI-IT-MS), and Hydrophilic Interaction Liquid Chromatography-Electrospray Ionization-Triple Quadrupole-Mass Spectrometry (HILIC-ESI-QqQ-MS). The recorded raw data were converted into a netCDF format using DataAnalysis 3.0 (Bruker Daltonik, Ettlingen, Germany) and imported into a MZmine 2.14 software (https://mzmine-2.soft112.com/) for automatic mass detection, chromatogram building and deconvolution into single extracted ions. Lipid mass spectrometry prediction together with tandem mass spectrometry data were used for molecular identification. PC, DGTA, DGTS and SM lipids were identified and quantified in the dataset obtained in positive ion mode, while PE, PG, PI and SQDG lipids were identified and quantified in the dataset obtained in negative ion mode.

The unsaturation index (UI) was calculated for each lipid class (y) as:

UI_y_ = [*Σ* (area % lipid_x_ × total number of double bonds lipid_x_)]/100

where lipid_x_ represents each single molecular species belonging to the y lipid class.

The average chain length (ACL) of each lipid class (y) was calculated using the formula:

ACL_y_ = [*Σ* (area % lipid_x_ × total number of acyl chains -C-atoms of lipid_x_)]/100

### 2.6 Analysis of fatty acids

Fatty acids (FAs) were separated and quantified by Gas Chromatography-Mass Spectrometry/Flame Ionization Detector (GC-MS/FID) analysis. They were first converted into the more volatile fatty acid methyl esters (FAMEs) by transesterification carried out in acidic conditions considering the presence of ether-linked glycerophospholipids and sphingomyelins which are usually not esterified under mild-basic conditions (Christie, 1993). Aliquots of 100 μl of crude cell extract were mixed with 500 μl of 1 M H_2_SO_4_ solution in dry methanol and left to react overnight at 50 °C. The reaction was then stopped and neutralized by adding 1 M NaOH. FAMEs were extracted three times with 500 μl of *n*-hexane and concentrated to 100 μl under nitrogen flux. Aliquots of 1 μl were then injected in splitless mode into a Thermo Scientific Trace GC-dual stage quadrupole II mass spectrometer (Thermo Fisher Scientific Inc., Waltham, MA) equipped with a Flame Ionization Detector controlled by the Xcalibur 2.0.7 software (Thermo Fisher Scientific Inc., Waltham, MA, USA). Gas chromatography was carried out using two DB-WAX capillary columns (30 m x 0.25 mm x 0.15 μm; Agilent Technologies, Santa Clara, CA, USA). The carrier gas was helium flowing constantly at 1.4 ml/min. The injector and MS transfer line temperatures were set at 250 °C, with a starting oven temperature of 50 °C progressively increased to 175 °C with steps of 25 °C/min, and to 230 °C with steps of 1 °C/min. The mass spectrometer quadrupole temperature was set at 150 °C and the source set at 230 °C. Electron impact mass spectra in positive ion mode were acquired at 70 eV, with scan runs covering a m/z range of 50–650. FAMEs were quantified using an external calibration curve generated with the FIM-FAME-7 standard mixture (Matreya LLC, Pleasant Gap, PA, USA). Fatty acid identification was confirmed by comparison with authentic standard compounds. When standards were not available, the fatty acid molecular formula was determined by electron ionization-MS and comparison of the obtained spectra with those available in the National Institute of Standards and Technology (NIST) database.

Throughout the text, fatty acids are designated according to the number of carbon atoms and double bonds in the acyl chain, using the abbreviation C:D (ω-x), where C is the number of carbon atoms, D is the number of double bonds, and X is the position of the first double bond from the methyl end (omega end). For example, stearic acid is designated as C18:0 (18 carbons, zero double bonds), whereas arachidonic acid is designated as C20:4 (ω-6) (20 carbons, four double bonds, first double bond at C6 from the methyl end).

## 3 Results

### 3.1 Quantitative NMR analysis

A preliminary comparative ^1^H NMR analysis of the membrane preparations from the four *Euplotes* species provided initial evidence for a common more pronounced commitment of the two polar species to construct their membranes with a maximum use of GPL molecules and a minimum use of BL molecules. In *E. focardii* and *E. nobilii*, PC plus PE molecules accounted for 96.7% and 99.4%, respectively, of the total membrane lipids, while BL (plus other structurally undetermined) molecules were quantified from 0.6% (*E. nobilii*) to 3.3% (*E. focardii*). Instead, they covered from 87.7% to 94.8% of the total membrane lipids in *E. crassus* and *E. raikovi*, respectively, while BL (plus other structurally undetermined) molecules varied from 12.3% (*E. crassus*) to 5.2% (*E. raikovi*).

**Figure 1:**
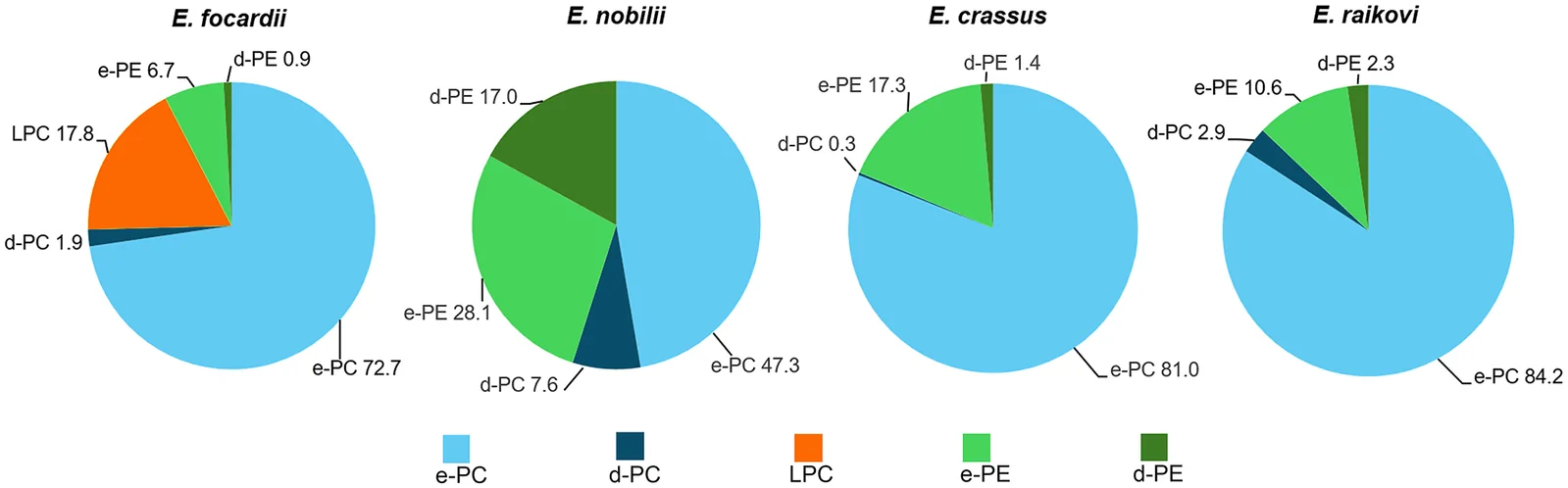
Relative molar ratios (expressed as % of the total GPL molecular pool) of diacyl (d), ether (e) and lyso (L) PC and PE forms, as determined by ^31^P NMR in the membranes of the four indicated *Euplotes* species.

A successive ^31^P NMR resolution of the ether (e), diacyl (d), and lyso (L) structural forms of the PC and PE fractions added evidence for significant quantitative differences in the membrane lipid composition not only between the two polar and the two temperate-water species, but also between one and the other polar species. As shown in Figure 1, *E. focardii* resulted to be unique for membranes containing the LPC form of GPL molecules (17.8% of the total), and *E. nobilii* unique for membranes containing the e-PC form in a percentage reduced to less than half (47.3%) of total GPL molecules, along with relatively abundant percentages of the d-PC, d-PE and e-PE forms (7.6%, 17.0% and 28.1% of total GPL molecules, respectively). On the other hand, *E. crassus* and *E. raikovi* revealed a GPL composition of their membrane lipids that was closely comparable in relation to the e-PC (81.0 and 84.2%, respectively), e-PE (17.3 and 10.6%, respectively) and d-PE (1.4 and 2.3%, respectively) forms, and appreciably different only in relation to the d-PC form (0.3 and 2.9%, respectively).

### 3.2 Qualitative LC-MS analysis

**Figure 2:**
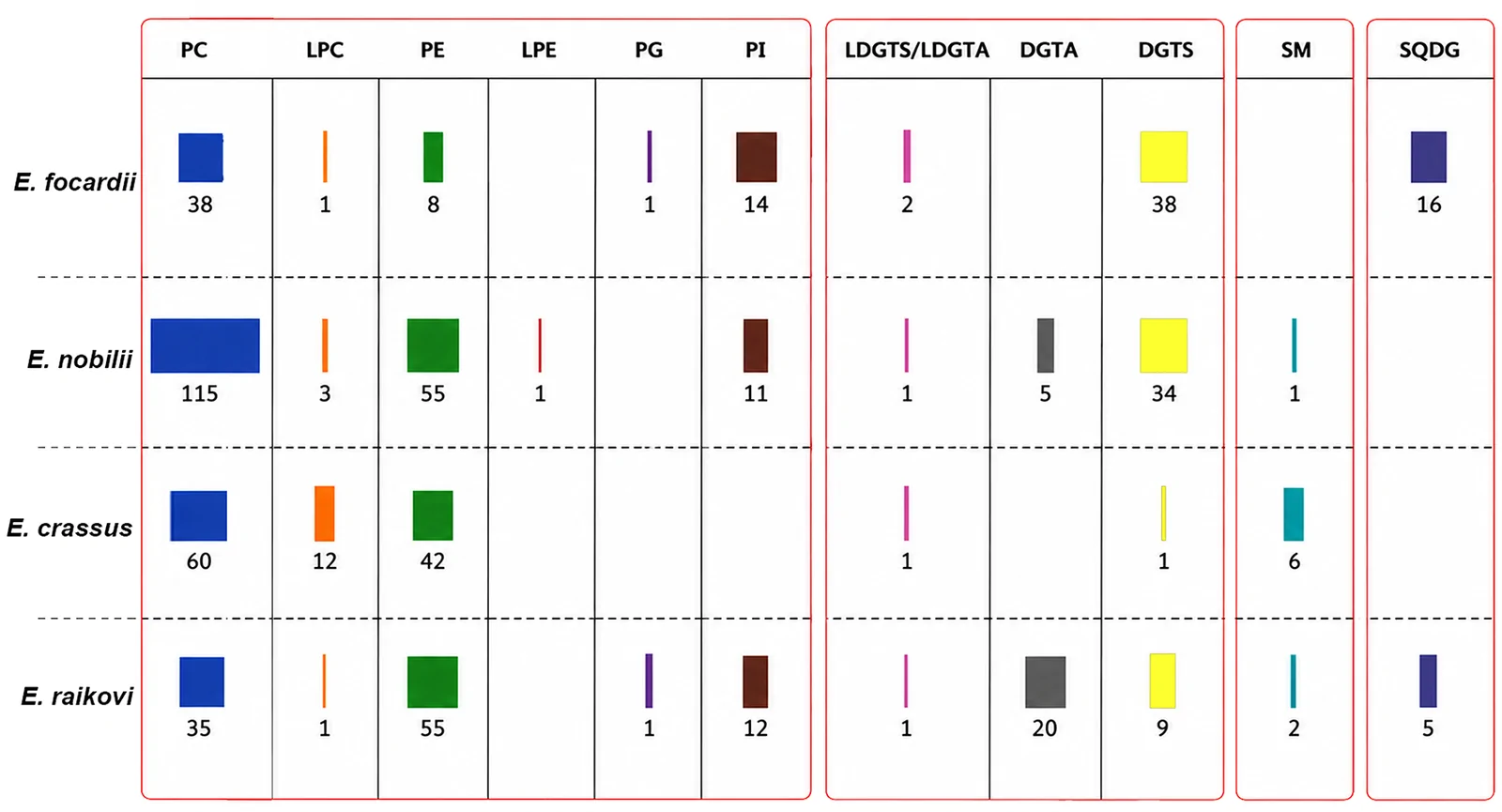
Number and distribution of lipid molecular species in the membranes of the four indicated *Euplotes* species. Columns are related to the lipid classes, and rows to the number of molecular species identified in each class.

A third qualitative, LC-MS based analysis of the membrane lipid composition was directed to determine the complete sets of molecular species representing the PC, PE and BL classes plus other lipid classes (LPE, PI, PG, SM, LDGTS/DGTA and SQDG) not detected by the two previous quantitative NMR analyses. As shown in Figure 2, two lines of evidence were obtained. First, the interspecific variations in the presence vs. absence of a given molecular class in the membrane composition appear to bear not significantly on the ecologically adaptive affinities linking *E. focardii* to *E. nobilii* and *E. crassus* to *E. raikovi*, but rather reflect the closer phylogenetic affinities linking *E. focardii* to *E. crassus*, and *E. nobilii* to *E. raikovi*. Emblematic is the case of the DGTA molecules detected in the latter two species, and not detected in the former two.

Second, the number of molecular species within each lipid class varies independently of the relative abundance of the class itself, and marked variations in the molecular composition of the GPL classes clearly distinguish the two polar species from one another. While *E. focardii* is unique for (i) a LPC class including only one molecular species that accounts for 17.8% of total GPL molecules and (ii) poorly represented PC and PE classes (38 and eight molecular species, respectively), *E. nobilii* is unique for largely represented PC and PE classes (115 and 55 molecular species, respectively). On the other hand, the two temperate-water species were similarly characterized by more balanced numbers of PC and PE molecular species (60 and 42, respectively in *E. crassus;* 35 and 55, respectively in *E. raikovi*).

### 3.3 Unsaturation index (UI) and average chain length (ACL)

**Figure 3:**
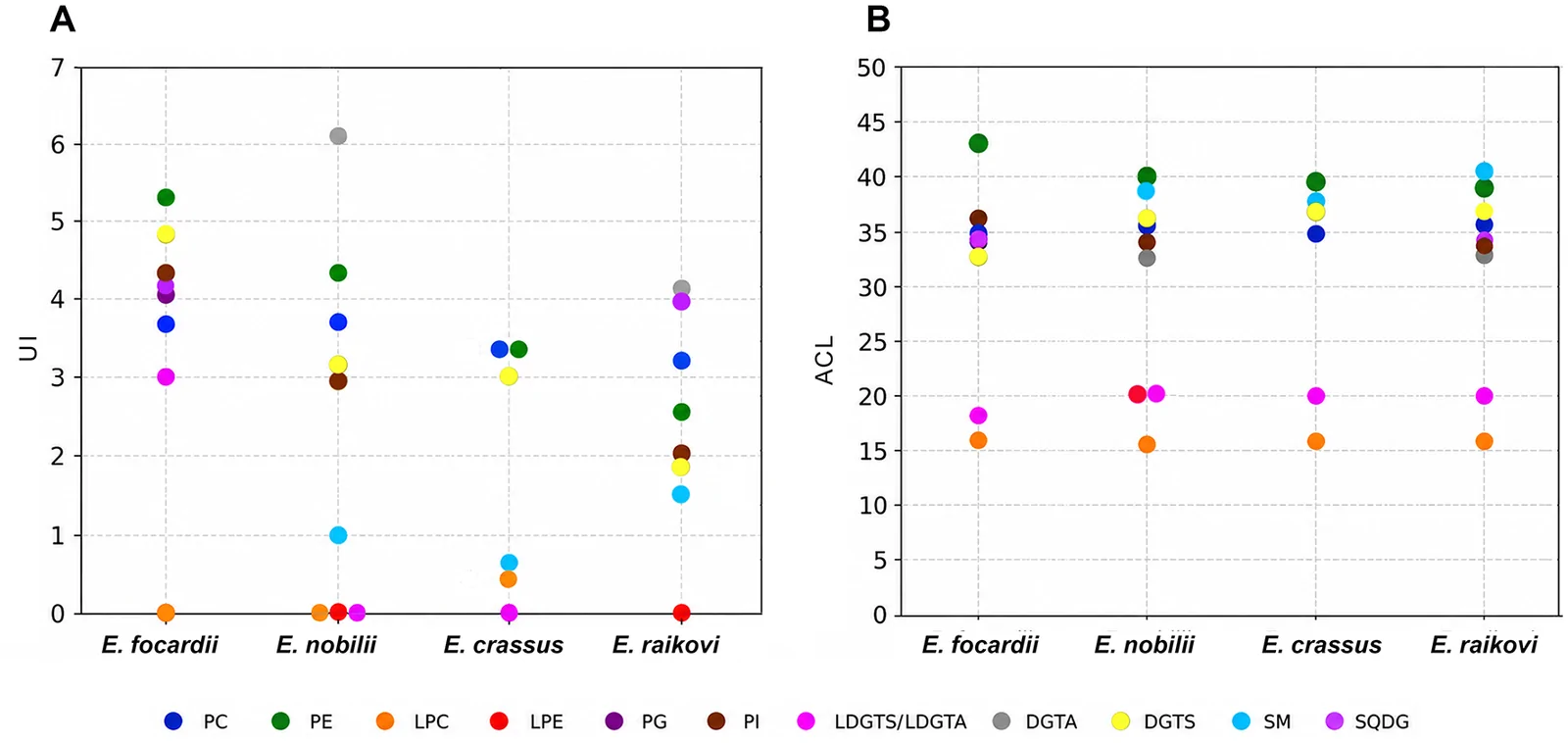
Unsaturation index (UI) and average chain length (ACL) values (**A** and **B**, respectively), measured for each lipid class in each of the four indicated *Euplotes* species.

To assess inter-specific variations in the degree of the membrane homeoviscous adaptation, the average number of double bonds was next calculated for each lipid class on the basis of the class relative UI value. As shown in Figure 3A (and in Supplementary Table S1), the UI values measured in *E. focardii* and *E. nobilii* membranes closely reflect a cold-adaptation effect resulting significantly higher than in *E. crassus* and *E. raikovi* membranes. With the exception of the LPC class in *E. focardii* and the poorly represented LPE, SM and LDGTS/LDGTA classes in *E. nobilii*, the UI values of all the other lipid classes similarly fall in a 3 to 6 range in the two polar species, while ranging from 0 to 4 in the two temperate water ones. In contrast with the UI value analysis, a comparative analysis of the lipid class ACL values resulted in mostly ambiguous inter-specific variations (Figure 3B and Supplementary Table S1).

### 3.4 GC-MS/FID fatty acid analysis

**Figure 4:**
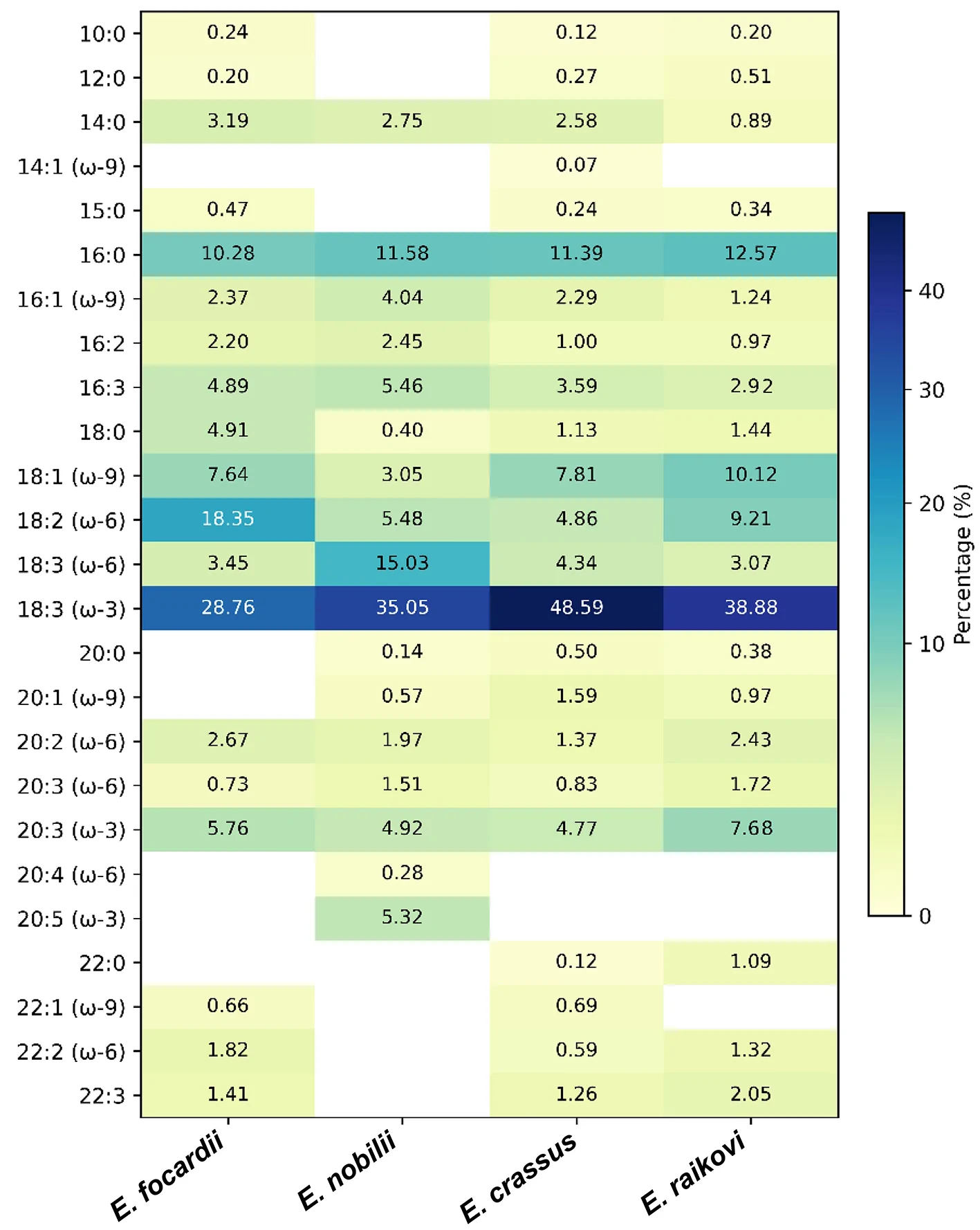
Heatmap of fatty acid (FA) composition, reported as percentage, in the four indicated *Euplotes* species. The percentages are represented by a scale of color intensity ranging from white (0%) to dark blue (50%). The position of double bonds is reported in parentheses when confirmed by comparison with standard compounds. When standard compounds were not available, the FA molecular formulas were determined by electron impact-mass spectrometry and comparing the spectra with those available from the NIST database.

Similarly in the membranes of the four *Euplotes* species, FA molecules with 16 and 18 C-atoms represent the predominant component, with the short-chain ( ≤ C15) and long-chain (C20–C22) FA molecules resulting poorly represented or absent at all. As shown in Figure 4, linolenic acid 18:3 (ω-3) was identified as the by far more abundant polyunsaturated FA component, ranging from a minimum of 28.76% in *E. focardii* to a maximum of 48.59% in *E. crassus*, while saturated FA molecules were primarily represented by palmitic acid 16:0. With respect to *E. crassus* and *E. raikovi*, in the two polar species FA molecules show quantitative differences in both the position of double bonds and composition of the long-chain PUFA component, with high levels of ω-6 PUFA molecules mainly represented by linoleic acid 18:2 (ω-6) in *E. focardii*, (18.4%) and linolenic acid 18:3 (ω-6) in *E. nobilii* (15%). Nevertheless, *E. nobilii* membranes differ from *E. focardii* membranes for a narrower FA range, restricted to C16–C20 chain lengths, and the unique presence of the highly unsaturated long-chain arachidonic acid 20:4 (ω-6) and eicosapentaenoic acid 20:5 (ω-3).

## 4 Discussion

Ciliates are ancient colonizers of the ecologically most diversified habitats, and their evolutionary success in this colonization clearly argues for an exceptional capability to promptly adapt the lipid composition of their membrane structure to effectively face any new environmental parameter. Seeking into the adaptive evolution of this capability has so far raised rather occasional research interest, essentially focused on the diversification of the lipid membrane composition in freshwater species of *Paramecium* (Kaneshiro, 1987) and *Tetrahymena* (Nozawa, 2011) as compared with marine species of *Parauronema* (Sul and Erwin, 1997), *Pleuronema* and *Fabrea* (Harvey et al., 1997). These five genera have distant phylogenetic relationships (Gao et al., 2016), and this distance makes equivocal to distinguish variations in the lipid membrane composition that have a genuine adaptive significance from variations that, instead, merely reflect remote phylogenetic relationships. By narrowing the comparison of the membrane lipid composition between polar and temperate water congeneric marine species of the same genus, *Euplotes*, we minimized this ambiguity providing a reliable experimental ground for identifying specificities of the composition that are consistent not only with the cold-adaptation of the species, but also with its eco-phylogenetic history.

As commonly observed in marine organisms from bacteria (Frolova et al., 2000; Goldfine, 2022; Yamashita et al., 2023) to animals (Chapelle, 1987; Jeong et al., 1996; Kraffe et al., 2004; Magnusson and Haraldsson, 2011; Lordan et al., 2017), the four studied *Euplotes* species have similarly revealed cellular membranes in which ether-linked GPL and DGTS molecules represent the two by far major structural lipid components. On the one side, the GPL component is amply documented to play key functions in stabilizing and protecting the membrane structure against a wide variety of oxidative, chemical and enzymatic stresses (Hazel, 1995; Hazel and Williams, 1990; Nagan and Zoeller, 2001; Kraffe et al., 2004; Dean and Lodhi, 2018). On the other side, the DGTS component, yet subject to wider interspecific variations in the relative concentrations, is generally regarded as functionally important in assisting organisms living exposed to particular conditions of phosphorus limitation to preserve a full functionality of their membranes by variously replacing GPL molecules (Kates, 1986; Grimont and Grimont, 2011; Martin et al., 2011; Sebastián et al., 2016).

While in the two *Euplotes* temperate water species the membrane lipid composition resulted to be largely similar in molecular diversity and relative concentration of various lipid classes, the lipidic composition of the *E. focardii* and *E. nobilii* membranes was characterized by a combination of common and species-specific cold-adaptive traits.

The most obvious common trait relates to the UI values similarly ranging from 3 to 6 in the lipid classes of the *E. focardii* and *E. nobilii* membranes (vs. a 0 to 4 range recorded in the two temperate-water species). A such higher range of UI values is fully in line with the general principle of membrane homeoviscous adaptation which applies to practically every organism that, called to adapt to drastic drops in the environmental temperature, needs to modify the membrane fluidity by increasing the degree of unsaturation of the lipid component (Renne and Ernst, 2023).

The species-specific cold-adaptive traits basically reside in the markedly different degrees of GPL molecular diversity that distinguish the *E. nobilii* and *E. focardii* membranes. Including a total of 185 GPL molecular species with 55 that are represented by PE molecules and account for approximately 45% of the entire GPL molecular pool, *E. nobilii* membranes clearly denote to have primarily invested on this lipid component for a successful cold-adaptation. In addition to acting on the degree of membrane fluidity through their highly disordered acyl chains (Dawaliby et al., 2016; Rosenberger and Marsh, 2011), PE molecules may as well promote membrane flexibility by utilizing their cone-shape geometry to generate a negative membrane curvature and the formation of non-bilayer phases (Bouchet et al., 2009; Lessen et al., 2022). The PE molecule regulatory function in the mechanism of membrane homeocurvature adaptation has more directly been documented in species of deep-sea ctenophores which, to successfully face the high hydrostatic pressure and low temperature of the abyssal habitat, optimize the curvature and packing of their membranes by primarily relying on an effective regulation of the molecular geometry and concentration of the PE lipidic component (Winnikoff et al., 2024; Winnikoff and Budin, 2025).

Including a total of only 62 GPL species (vs. the 185 species detected in *E. nobilii*), the *E. focardii* membranes are characterized by a rather homogeneous lipid composition, and this homogeneity is further magnified by the presence of a single LPC molecular species that accounts for 17.8% of the total GPL molecular pool. As for the PE component in the *E. nobilii* membranes, this unusually high concentration of LPC molecules in the membranes of *E. focardii* clearly implies a central role played by these molecules in determining the membrane biophysical properties and organization. The effects might depend not only on the LPC molecular concentration, but also on the interactions of these molecules with the surrounding lipid matrix. Due to their inverted-cone molecular geometry, LPC molecules are more commonly known to promote a positive membrane curvature (Mohandas et al., 1978; Koynova and Caffrey, 1998; Liu et al., 2026). However, included in a suitable lipidic environment, they can as well induce the formation of partially or fully interdigitated membrane domains which, being characterized by a tighter lipid packing and a reduced membrane thickness, would be responsible for local decreases of the membrane fluidity (Slater and Huang, 1988; Lu et al., 1997, 2001). The coexistence in the *E. focardii* membranes of a high LPC molecular concentration with a highly polyunsaturated lipid environment implies an interplay of complementary, yet potentially antagonistic, mechanisms making cold-adaptation of these membranes the result of a fine balance between membrane flexibility and structural stability. While polyunsaturated components would work for a general enhancement of the membrane fluidity by reducing lipid packing, LPC molecules might promote local ordering and curvature remodeling.

In conclusion, evidence has been provided that in *Euplotes* polar species cold-adaptation of the membrane lipid composition reflects differences in the species evolutionary histories and timescales of colonization of the polar habitat. In *E. focardii*, it is consistent with a strictly psychrophilic behavior and an evolutionarily ancient (endemic) necessity to cope with the persistently sub-zero temperatures of the Antarctic waters. Instead, in *E. nobilii*, it is consistent with a more flexible psychrotrophic behavior and the necessity to cope with a more fluctuating range of cold temperatures that this bipolar species meets in migrating from one to the opposite pole via the deep ocean currents.

## Data Availability

The original contributions presented in the study are included in the article/Supplementary material, further inquiries can be directed to the corresponding author.
