# Supplementary material for: Cold-adaptive variations in the membrane lipid composition in polar species of the ciliate Euplotes
Source: Front Microbiol. 2026 Aug 24;17:1909674. doi: 10.3389/fmicb.2026.1909674 (PMC13548811; doi:10.3389/fmicb.2026.1909674)
Supplement: Supplementary file 1 [file Data_Sheet_1.pdf]

Supplementary Material

Supplementary Table S1. List of Unsaturation Index (A) and Average Chain Length (B) calculated for each lipid class in the indicated *Euplotes* species.

**A**	PC	LPC	PE	LPE	PG	PI	SM	LDGTS/LDGTA	DGTA	DGTS	SQDG
E. focardii	3,7	0	5,33		4	4,26		3		4,9	4,14
E. nobilii	3,73	0	4,29	0		3,11	1	0	6,15	3,16	
E. crassus	3,37	0,39	3,37				0,66	0		3	
E. raikovi	3,19	0	2,56			2,04	1,6	0	4,14	1,92	3,94

**B**	PC	LPC	PE	LPE	PG	PI	SM	LDGTS/LDGTA	DGTA	DGTS	SQDG
E, focardii	34,73	16	43,1		34	35,61		18		33,84	34,5
E, nobilii	35,6	15,66	40,28	20		34,36	38	20	33,88	36,22	
E, crassus	34,93	16,04	39,77				38,39	20		38	
E, raikovi	35,54	16	39,22			34,25	40,6	20	33,69	36,91	34
